# Trait Variations and Probability Grading Index System on Leaf-Related Traits of *Eucommia ulmoides* Oliver Germplasm

**DOI:** 10.3390/plants10112280

**Published:** 2021-10-25

**Authors:** Peng Deng, Xiangchen Xie, Feiyu Long, Liang Zhang, Yonghang Li, Zhangxu Zhao, Shiyao Yang, Yiran Wang, Ruishen Fan, Zhouqi Li

**Affiliations:** 1 College of Forestry, Northwest A&F University, Xianyang 712100, China; dengpeng@nwafu.edu.cn (P.D.); xxc@nwafu.edu.cn (X.X.); fly179@nwafu.edu.cn (F.L.); zhangliang2017@nwafu.edu.cn (L.Z.); yonghang@nwsuaf.edu.cn (Y.L.); wyr08@nwafu.edu.cn (Y.W.); fanruishen@nwsuaf.edu.cn (R.F.); 2 College of Economics and Management, Northwest A&F University, Xianyang 712100, China; zhaozhangxu@nwafu.edu.cn; 3 College of Life Sciences, Northwest A&F University, Xianyang 712100, China; monster@nwafu.edu.cn

**Keywords:** *Eucommia ulmoides* Oliver, trait variations, probability grading, quantitative traits, planting models, leaves

## Abstract

*Eucommia ulmoides* Oliver (EUO), an economic tree grown specifically in China, is widely used in various fields. To satisfy the requirements of industrial development, superior varieties need to be selected for different uses. However, there is no unified standard for breeders to reference. In this study, leaf-related traits were classified by a probability grading method. The results indicated there were significant differences between different planting models for the studied traits, and the traits in the Arbor forest model showed more abundant variation. Compared with genotype, the planting model accounted for relatively bigger variance, indicating that the standard should be divided according to planting models. Furthermore, the optimum planting model for different traits would be obtained by analyzing the variation range. Association analyses were conducted among traits to select the crucial evaluation indexes. The indexes were divided into three grades in different planting models. The evaluation system on leaf-related traits of EUO germplasm was established preliminarily, which considered planting models and stability across years for the first time. It can be treated as a reference to identify and evaluate EUO germplasm resources. Additionally, the study served as an example for the classification of quantitative traits in other economically important perennial plants.

## 1. Introduction

*Eucommia ulmoiudes* Oliver (EUO) is an economically important tree belonging to the monotypic family Eucommiaceae [1]. Unique to China, it has a high value for development and utilization. EUO has been used in China for more than 2000 years as a traditional Chinese medicine [2,3]. To date, 132 chemical compounds have been identified from it [4], and some of them contribute to treating hypertension, hyperlipemia, Alzheimer’s disease, and aging [5]. Due to their health care function, the leaf and the bark were listed in the “Pharmacopia of China” from 2005 [6]. Surprisingly, the oil extracted from its cotyledon contains 66.4% of α-linolenic acid, 8–60 times greater than that in Oliver oil [7]. Additionally, EUO is a widely distributed tree species producing *Eucommia* rubber (*Eu*-rubber), a trans-polyisoprene (TPI) [8]. TPI performs better in insulation and corrosion resistance compared with cis-polyisoprene (CPI) produced by *Hevea brassiliensis* [9], which led to its use in insulated cables and medical instruments [10]. EUO is widely cultivated in 27 provinces in China, with a cultivation area of 0.35 million hectares [11]. Its better physical characteristics and extensive distribution make it a promising alternative or supplementary resource to *Hevea brassiliensis* [10,12]. A study reported that EUO orchards can produce 4000 kg fruit per hectare, about 520 kg of TPI, and the input–output ratio can reach up to 646.49% [7]. Furthermore, it is also used for landscaping and soil and water conservation as its peculiar fruit and a stronger adaption to the environment, respectively [13]. Therefore, it is called the “Chinese sacred tree” or “plants gold” with economic, social, and ecological benefits.

Almost all parts of EUO, including the bark, branches, leaves, flowers, and fruit, are of high value in various fields [14,15,16,17]. Compared with other organs, leaves are easy to access and abundant in yield [14]. They can be harvested throughout the entire growing season and are unconstrained by the reproductive growth stage. Therefore, it is an ideal tissue type to exploit. To date, leaves of EUO have been used in numerous fields. To satisfy the requirements of different industries, superior varieties or clones need to be selected according to target traits. For leaves, many traits are quantitative characteristics, and evaluation criteria are needed as a reference for breeders. However, the existing classification standard of leaf-related traits is not clear or comprehensive, which restricts the identification and evaluation of EUO germplasm.

There are various grading methods for the classification of quantitative traits, which include average classification, clustering analysis, and probability grading. Traditionally, the grading method has equal differences based on experience. Simple and feasible as it is, it cannot reflect the distribution of traits. Furthermore, it is difficult to reach an agreement [18]. Clustering analysis is utilized to classify the study objects into several clusters according to the similarities among them. This fits research objectives with large differences. For example, this method has been used to classify daylily flower color [19]. However, the effect of grading strongly depends on the study objects. Specifically, the sample must be representative and the sample capacity should be large enough to ensure the reliability of the standard. For example, a total of 36,000 *Panax notoginseng* seedlings were collected from 30 major producing areas to establish the grading standard of seedlings [20]. For the classification of the morphological traits, geometric morphometric analysis is more precise in describing the shape of leaves [21]. Additionally, the leaf shape fractal dimension (FD) is considered as the best index to reflect the complexity of leaf shape. However, it is more complicated and cannot be used for the other traits. A study reported that there was strong correlation between the FD and length-to-width ratio. Therefore, sufficient information on leaf shape can be provided by the length-to-width ratio, the simpler index [22]. Probability grading is an approach based on the probability distribution feature of quantitative trait values. It illustrates the average level and dispersion degree of traits and reflects the systematic position of the individual in the overall level [23]. Due to the objectivity in description, it contributes to the unification of standards among different breeders [18]. There are 3-grade and 5-grade standards for it [18,23]. Considering that the distribution of quantitative traits in nature obeys normal distribution [23], which shows the distribution characteristics of larger in the middle and smaller at both ends, the middle grade is allocated 40% of the occurrence probability. In addition, the values of middle grade are distributed around the average. Therefore, the probability grading method has a greater guidance value in practice. To date, it has been successfully used in numerous economically important trees, and ideal effects were obtained. Liu [24] proposed the probability grading method for the first time and successfully used it in the classification of economic traits of peach trees. Soon afterwards, Liu [23] and Liu [18] developed the method and applied it to the evaluation of quantitative traits in Chinese Jujuba. In recent years, the method has been used in the classification of important quantitative characteristics in mango [25], *Armeniaca vulgaris* [26], table grape [27], wild *Actinidia eriantha* [28], and apple fruit [29], among others. Surprisingly, it was also used to define the shape of the ray floret in large-flowered chrysanthemum to make the standard more accurate and objective than direct observation [30]. For EUO, it has been used in the classification of quantitative traits in male flowers [31] and fruit [32], but similar studies are rare in leaves of EUO. Studies on the classification of leaf-related traits of EUO mainly focused on the secondary metabolites of leaves [33,34], which is clearly not enough for the utilization of EUO. A more comprehensive probability grading system based on target traits is necessary.

EUO is a woody perennial plant; it usually takes 7 years after planting before it flowers and fruits [35], and then it turns from the vegetative to the reproductive growth stage. Unlike other generative organs, leaves can be obtained in all growing stages. Previous studies have reported significant differences in leaf-related traits between stages not only in EUO [36,37,38], but also in other species [39,40,41]. There are two kinds of planting models for EUO in production, including the Leaf-oriented cultivation model (LCM) and Arbor forest model (AFM) [42]. The difference between them in essence is that they are, respectively, in the vegetative and reproductive growth stages. To better guide production, the classification system should be formulated according to planting models. 

In this study, the probability grading method was used to set up a standard for leaf-related traits of EUO. To satisfy the requirements of different industries, traits were classified according to target traits. The variations and distribution types of traits in different planting models were analyzed. In addition, this study revealed the impacts of planting models, genotypes, and tree age on different traits. To remove duplicated traits in evaluating germplasm, association analyses were analyzed. Considering the limited germplasm resources and generality of the standard, evaluation indexes were classified into three grades in separate models. This could serve as a reference for the evaluation, selection, and discrimination of EUO germplasm, which is beneficial for accelerating the process of EUO breeding.

## 2. Materials and Methods

### 2.1. Plant Materials

In this study, 56 Arbor forest model (AFM) trees and 48 Leaf-oriented cultivation model (LCM) clones were employed. Among these, 56 AFM trees comprised 15 EUO cultivars and 41 superior individuals. The 15 EUO cultivars were selected from different areas of China [43], and the 41 superior individuals were selected from the F1 generation of cross breeding of the superior cultivars according to the tree height and ground diameter. Forty-four in 56 of AFM trees and “Qinzhong NO. 1–4” were grafted to 48 LCM clones in August 2018. A field trial was set up at the nursery of the College of Forestry at Northwest Agricultural and Forestry University (108°05′ E, 34°24′ W) in Yangling, China. The 56 AFM trees were germinated in the EUO Germplasm Resources Collection Area at a spacing of 2 m × 2 m in March 2010. In addition, 48 LCM clones were laid out in randomized complete blocks at a spacing of 0.5 m × 0.5 m, including three blocks with 48 clones per block. There were 90 seedlings for each clone, with 30 seedlings in each block. 

### 2.2. Studied Traits

The leaf-related traits of EUO in this study are listed in Table 1. Among these, T1 to T7 were used to describe the leaf morphology, and they can be used to distinguish and identify germplasms [44,45,46]. There are two main harvest products in *Eucommia ulmoides* Oliver: gutta-percha and chlorogenic acid. The total economic output depends on their contents and leaf yield. T8 and T9 depicted leaf yield, and T10 to T11 showed the content of secondary metabolites in leaves. Additionally, adaption must be considered when they are planted in different areas. T12 to T15 represented the water status of leaves, which were used to measure drought resistance [47,48,49].

### 2.3. Planting Models

**Figure 1:**
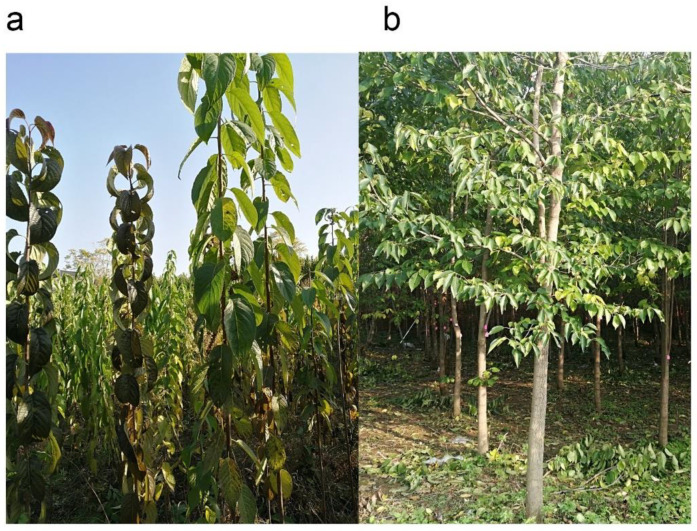
Leaves in different planting models, including (**a**) the Leaf-oriented cultivation model and (**b**) the Arbor forest model.

The Leaf-oriented cultivation model (Figure 1a) and Arbor forest model (Figure 1b) of *Eucommia ulmoides* Oliver trees were displayed as follows.

### 2.4. Measurement of Leaf Water Status

For each LCM clone, a fresh sunshine leaf (the 8th leaf from the top) was taken from a healthy individual selected randomly from each block in August 2019. For every AFM tree, a fresh sunshine leaf (the 8th leaf from the top) was collected from similar-size branches in the direction of north, southeast, and southwest in August 2019. Thus, there were 3 replicates for every LCM clone or AFM tree. Leaves were collected in the evening and placed in water to absorb it throughout the night. The fresh leaf was weighed (W_f_) and fastened to the PMS Model 1000 Pressure Chamber Instrument (PMS Instrument Company, Albany, OR, USA). By gradually applying pressure and recording the pressure and the weight of removed water, a fitted curve and straight line were acquired. The leaf was placed in a drying oven, and the dry weight (W_d_) was recorded [47]. 

### 2.5. Sample Collections and Measurement of Leaf Morphological Traits

For each LCM clone, fresh leaves (the 10th leaf from the top) were collected from 10 healthy seedlings selected randomly from each block in September 2019, and the total fresh weight of them was about 20 g. For every AFM tree, the fresh leaves (the 10–15th leaf from the top) were collected from similar-size branches in the direction of north, southeast, and southwest in September 2017, 2018, and 2019, and the total fresh weight of them was about 60 g.

Leaf area (LA), leaf length (LL), and leaf width (LW) were measured by a Yaxin-1241 leaf area meter (Beijing Yaxin Technology Co., Ltd., Beijing, China). Petiole length (PL) and the thickness of a single leaf (TL) were measured using a vernier caliper. The single leaf weight (WL) was measured using an electronic balance. 

### 2.6. Sample Processing and Measurement of the Secondary Metabolite Content in Leaves

The samples were placed in a drying oven at 60 °C. Then, they were stored at room temperature for later use. The samples were ground into powder using a DHS TL2020 tissue grinder apparatus (DHS Life Science & Technology, Beijing, China) before measurement.

The extraction and determination of gutta-percha were performed following the description of *Ma* [50]. Briefly, 100 mL NaOH solution (10%, *m*/*m*) was added to 5 g of powder in a water bath at 90 °C for 3 h, twice. After filtration, 60 mL HCI was added to the residue in a water bath at 40 °C for 2 h. Then, about 60 mL ethanol (60%, *v*/*v*) was added to the residue after filtration The solution was incubated for 1h and put into an ultrasonic cleaner (40 kHz, 40 °C) for 0.5 h. The content of gutta-percha (GP) could be obtained after filtration and drying at room temperature.

The extraction of chlorogenic acid was conducted with reference to Dong [51]. Separation and determination were performed using an Agilent Technologies (Santa Clara, CA, USA) HPLC system model 1260 following Ye [52]. The detection of chlorogenic acid was recorded at 320 nm. The 1260 DAD-chemstation (offline) software was utilized for data analysis (using peak area values) and the content of chlorogenic acid (CA) was determined using external calibration. The chlorogenic acid standard substance (HPLC ≥ 98%) was purchased from Shanghai Yuanye Bio-technology Co., Ltd (Shanghai, China).

### 2.7. Data Analyses

#### 2.7.1. Chlorogenic Acid Content Determination

The chlorogenic acid content was determined by the following standard curve:

(1) $$y=2\times 10^{-5}x\;+\;0.0041\;(R^{2}=0.9992)$$

where *x* is the peak area and *y* is the content.

#### 2.7.2. Calculation of Water Status Parameters

According to the fitted curve and straight line, two formulas were obtained as follows:

(2) $$1/\psi_{w}=aV_{1}^{b}$$

(3) $$1/\psi_{\pi }=c+dV_{2}$$

In this way, the following parameters were obtained:

(4) $$\psi_{\pi }^{0}=\frac{1}{a}{\left(\frac{d}{ab}\right)}^{\left(\frac{b}{1-b}\right)}$$

(5) $$\psi_{\pi }^{100}=\frac{1}{c}$$

(6) $$V^{\prime }={\left(\frac{d}{ab}\right)}^{\left(\frac{1}{b-1}\right)}$$

(7) $$V_{0}=-c/d$$

(8) $$\;V_{t}=W_{s}-W_{d}$$

(9) $${\begin{array}{c} RWD \end{array}}_{0}=\;\left(V^{\prime }/V_{t}\right)\times 100\%$$

(10) $$Ma=\;\left(V_{t}-V_{0}\right)/V_{t}$$

*V’*: Water content of osmotic at turgor loss;
*V_0_*: Water content of osmotic at full turgor;
*Vt*: Tissue-saturated water content.

#### 2.7.3. Variance Analyses and Multiple Comparisons

Variance analyses and Tukey’s HSD multiple comparisons were conducted using SPSS 22.0.

#### 2.7.4. Normal Distribution Test and Grading Standard

The normal distribution test was performed using the “Shapiro test” protocol in R software. Traits incompletely obeying a normal distribution were treated as normal distribution only when the main part was normally distributed. Traits were separated into 3 grades by (*X* − 0.5246*S*) and (*X* + 0.5246*S*) if they obeyed a normal distribution (“*X*” indicates the mean and “*S*” indicates the standard deviation of the traits) [23]. 

## 3. Results

### 3.1. Leaf-Related Trait Variations

As shown in Table 2, leaf-related traits in the Arbor forest model showed a more abundant variation. It was likely that differences accumulated over time. For the Arbor forest model, the descending order of coefficient of variations (CV) were: leaf yield, secondary metabolite content, morphology, and the water status of leaves. The order of the Leaf-oriented cultivation model was similar to the Arbor forest model except for the order of the top two traits. The CV of secondary metabolite content was prominently larger than leaf yield.

For leaf morphological traits, whether in the Leaf-oriented cultivation model or the Arbor forest model, LA owned the largest range of variation, while NV owned the smallest. The CV of LA was 23.1 and 15.1% in the Arbor forest model and Leaf-oriented cultivation model, respectively. The counterpart of NV was 8.1 and 5.7% (Table 2).

For leaf yield traits, the WL contained broader variation (17.2%) in the Leaf-oriented cultivation model, while the TNL had broader variation (36.55%) in the Arbor forest model (Table 2). 

For secondary metabolite content, the CA was more extensive in variation (35.99% for the Arbor forest model, 33.14% for the Leaf-oriented cultivation model); however, the GP was much narrower (23.57 and 19.87%, respectively) (Table 2).

For leaf water status traits, the descending order of CV showed a consistent trend, whether in the Leaf-oriented cultivation model or the Arbor forest model. The descending order was as follows: *RWD*_0_, *ψ*_π_^0^, *ψ*_π_^100^, and *Ma*. The CVs ranged from 6.8 to 25.3% in the Arbor forest model, the counterpart of which was 5.9 to 16.6% in the Leaf-oriented cultivation model (Table 2).

The CV shows the genetic potential of traits [23], and it could be used to describe and distinguish germplasm resources [53]. Characteristics owning a large range of variation could be better used to classify germplasm resources. Additionally, the degree of variation determined the effectiveness of selection. The leaf yield and the secondary metabolite content owned larger variation whether in the Leaf-oriented cultivation model or Arbor forest model. Accordingly, more attention should be given to them in superior germplasm selection. 

### 3.2. Variance Analyses and Multiple Comparisons of Leaf-Related Traits

Table 3 indicates that there is a significant difference between different planting models for the studied traits. Compared with genotype, the planting model accounted for relatively bigger variance. Therefore, the grading standard should be divided according to planting models. For TL and leaf water status traits, there was a non-significant difference among genotypes. Considering their non-ideal effect in germplasm identification, they should be excluded in the evaluation system. On this basis, variance analyses were conducted among tree ages for the same trait. It showed there was a significant difference among the tree ages in different planting models, while differences were non-significant within the same planting model. Therefore, it was necessary to unify the standards in the same planting model. Furthermore, the optimum utilization model for different traits would be obtained by analyzing the variation range.

For the leaf morphological traits shown in Table 2, the leaves in the Leaf-oriented cultivation model were remarkably larger than those in the Arbor forest model. This was consistent with another study. Du [36] reported that the leaves became smaller in size with increasing tree age in EUO. Compared with the leaves in the Arbor forest model, the leaves in the Leaf-oriented cultivation model were easy to access, which could save labor costs. Therefore, the leaves in the Leaf-oriented cultivation model were more likely to satisfy the demand for leaf use.

Interestingly, the two secondary metabolites appeared to have different features between different planting models. For GP, the Arbor forest model had a higher content than the Leaf-oriented cultivation model. This was consistent with a previous study. Du [36] found that the content of GP tended to increase gradually with tree age. However, it was the opposite for CA. This was consistent with previous research. Yang [37] and Zhang [38] pointed out that the CA content was reduced along with the tree age. It seemed that the leaves in the Leaf-oriented cultivation model were more suitable for medicinal use, while those in the Arbor forest model were more fit for rubber use (Table 2).

Leaf water status can be used to evaluate drought resistance [49], among which the *ψ*_π_^0^ was considered as the best index to measure drought resistance [48] (p. 59). Many traits were negatively correlated with drought resistance, except *Ma*. The Arbor forest model had an advantage over the Leaf-oriented cultivation model in drought resistance (Table 2). Perhaps the seedlings in the Leaf-oriented cultivation model were more susceptible to the environment. In addition, these findings agreed with those of a preceding study [39]. Li found that *ψ*_π_^0^, *ψ*_π_^100^, and *RWD*_0_ decreased significantly with the increase in age in the platform for *Populus simonii,* which indicated an increase in drought resistance with tree age. 

In general, there was a significant difference between different planting models for all traits measured in this study. For traits in different models, the optimum utilization planting model can be obtained by analyzing the variation range. Consequently, the relationship between the target traits and planting models was expounded, which contributed to the improvement of EUO breeding. 

### 3.3. Association Analyses among Leaf-Related Traits

To reduce repetitive traits in evaluating EUO germplasm, association analyses were conducted among leaf-related traits in different planting models (Table 4). There were (extremely) significant correlations among LL, LW, LWR, WL, and LA in the two planting models, which indicated LA provided more information. In addition, LA had larger variation compared with NV and PL. Therefore, LA can be the best index to show leaf morphology. Unlike WL, TNL was a relatively independent index, which can be used to represent leaf yield. Gutta-percha and chlorogenic acid were the main harvest products, and GP and CA represented the effective constituent content. In conclusion, LA, TNL, GP, and CA can be the evaluation indexes.

### 3.4. Distribution Type of Leaf-Related Traits

As seen in Table 2, all traits were normally distributed. The test for a normal distribution is the precondition of probability grading; it can indicate whether the samples are representative. If the samples obey a normal distribution, the germplasm resources studied can be treated as the result of random sampling. In this way, they can represent the overall to some extent, and the standard formulated by the germplasm can be more effective. The distribution of quantitative traits in nature is in accordance with a normal distribution. The reason why some traits do not obey a normal distribution is directional selection in breeding [23]. In addition, limited germplasm can affect it to some extent. These will lead to some extreme points. Usually, they belong to the first or last grade of the total and can affect the distribution. However, the remaining part still obeys a normal distribution by removing the extreme values. A situation like this can be treated as a normal distribution [32]. In this way, the extreme values were divided into the first or last grade. 

### 3.5. Probability Classification of Leaf-Related Traits

**Figure 2:**
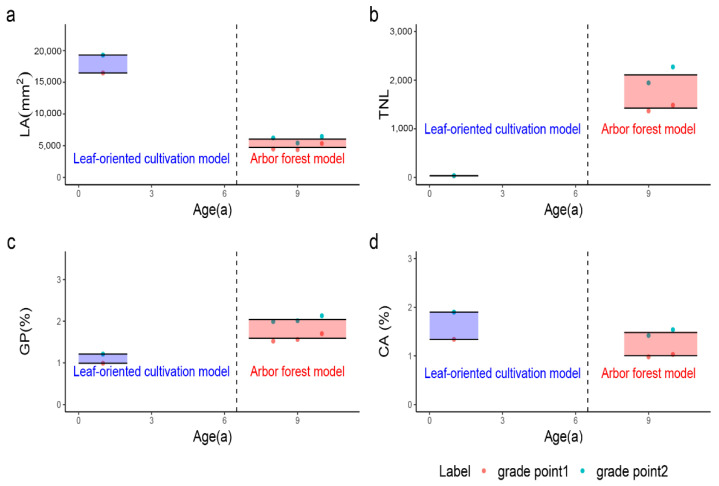
The classification criteria of the main evaluation indexes, including (**a**) LA, (**b**) TNL, (**c**) GP, and (**d**) CA. The black solid lines above and below the rectangle indicate the average level of grade points in each planting model. The lines at the top and bottom of the rectangle box represent the adjusted grade points. Traits were divided into three grades by the rectangle box: the 1st grade (the part below the rectangle), the 2nd grade (the part within the rectangle), and the 3rd grade (the part above the rectangle). The dashed line represents the boundary between the Leaf-oriented cultivation model and Arbor forest model.

As shown in Figure 2 and Table S1, considering the stability across the years, dynamic measurements were obtained from 2017 to 2019 for the quantitative traits in the Arbor forest model, which corresponded to 8a to 10a of the tree ages. Grading points in different years could be unified within the Arbor forest model, as a non-significant difference was observed. However, the utilization of the Leaf-oriented cultivation model in production mainly focuses on 1a seedlings by coppicing every year. To better guide production, the standard in the Leaf-oriented cultivation model was formulated by 1a seedings. To promote and apply the standard, it should be easy to remember. Therefore, it was necessary to adjust the grading points slightly. Special attention was required for the part that was adjusted no more than 0.1 SD for corresponding traits [23].

Table 5 shows that the evaluation indexes were classified into three grades, respectively, in different planting models. Grade 3 was better in quality, Grade 2 was medium, and Grade 1 was worse.

LA was used to distinguish leaf morphology. In the Leaf-oriented cultivation model, the variation range was 12,314.8–22,737.1 mm^2^, with an average of 17,877.1 mm^2^. The grading standard was as follows: grade 1 (smaller) ≤ 16,465 mm^2^, 16,465 mm^2^ < grade 2 (medium) < 19,230 mm^2^, grade 3 (bigger) ≥ 19,230 mm^2^; in the Arbor forest model, LA ranged from 1971.4 to 12,558.9 mm^2^, with an average of 5382.5 mm^2^. The classification criterion was as follows: grade 1 (smaller) ≤ 4730 mm^2^, 4730 mm^2^ < grade 2 (medium) < 6035 mm^2^, grade 3 (bigger) ≥ 6035 mm^2^.

TNL was used to represent leaf yield. In the Leaf-oriented cultivation model, the variation range was 24–50, with an average of 35. The grading standard was as follows: grade 1 (less) < 32, 32 ≤ grade 2 (medium) < 37, grade 3 (more) ≥ 37; in the Arbor forest model, the TNL ranged from 347 to 4060, with an average of 1767. The classification criterion was as follows: grade 1 (less) < 1430, 1430 ≤ grade 2 (medium) ≤ 2100, grade 3 (more) > 2100.

For GP, in the Leaf-oriented cultivation model, the variation range was 0.71–1.62%, with an average of 1.10%. The grading standard was as follows: grade 1 (lower) < 1.00%, 1.00% ≤ grade 2 (medium) ≤ 1.20%, grade 3 (higher) > 1.20%; in the Arbor forest model, the GP ranged from 0.67 to 3.29%, with an average of 1.82%. The classification criterion was as follows: grade 1 (lower) < 1.60%, 1.60% ≤ grade 2 (medium) < 2.05%, grade 3 (higher) ≥ 2.05%.

For CA, in the Leaf-oriented cultivation model, the variation range was 0.56–2.79%, with an average of 1.62%. The grading standard was as follows: grade 1 (lower) < 1.35%, 1.35% ≤ grade 2 (medium) < 1.90%, grade 3 (higher) ≥ 1.90%; in the Arbor forest model, the CA ranged from 0.30 to 2.35%, with an average of 1.25%. The classification criterion was as follows: grade 1 (lower) ≤ 1.00%, 1.00% < grade 2 (medium) < 1.50%, grade 3 (higher) ≥ 1.50%.

The quantitative traits were divided into five grades in most studies; the occurrence probability of Grades 1–5 was 10, 20, 40, 20, and 10%, respectively [30]. The selection of forest trees is a long-term process; it usually requires multiple selections, which is mainly due to the uncertainty of future performance. Therefore, the inclusion criterion should be reduced properly for forest tree selection. Therefore, it is appropriate to combine the first two and last two grades since the best grade is too narrow. In conclusion, it was preferable to divide the traits into three grades for this study; the occurrence frequency of Grades 1–3 was 30, 40, and 30%, respectively. 

The probability grading index system on leaf-related traits of EUO germplasm was established preliminarily. It can be a standard for breeders to reference. Superior germplasm with a higher leaf yield and effective constituent content can be selected according to different target traits. They can greatly promote the economic performance in certain areas, which is quite critical to the large-scale production of industries.

## 4. Conclusions

In this study, to identify and evaluate EUO germplasm, leaf-related traits were classified by a probability grading method according to target traits. There was a significant difference between different planting models for the studied traits, and the traits in the Arbor forest model owned abundant variations. The planting model accounted for a larger proportion of variance compared to the genotype, indicating the necessity to establish the grading standard according to planting models. Multiple comparisons analyses indicated there was a significant difference among the tree ages in different planting models, while a non-significant difference was observed within a planting model. Therefore, the grading points were unified in diverse years in the Arbor forest model. Considering the overlap among the traits in evaluating germplasm, association analyses were conducted to select the critical evaluation indexes. To make the standard more universal, the 3-grade standard was more suitable than the 5-grade standard. Germplasm in the Leaf-oriented cultivation model was more appropriate for leaf use and medical use, while germplasm in the Arbor forest model was more appropriate for rubber use and drought resistance. The evaluation system on leaf-related traits of EUO germplasm was established preliminarily, which considered target traits, planting models, and stability across the years. This can be a reference for the selection of superior germplasm for different target traits of EUO in different planting models. However, limited germplasm resources restrict the application of this standard to a wider scale. Therefore, additional studies should enlarge the germplasm resources.

## Figures and Tables

**Table 1:** Leaf-related traits of *Eucommia ulmoides* Oliver in the current study.

No.	Traits	Abbreviation
T1	Leaf length (mm)	LL
T2	Leaf width (mm)	LW
T3	Leaf area (mm^2^)	LA
T4	Leaf length–width ratio	LWR
T5	Thickness of a single leaf (mm)	TL
T6	Petiole length (mm)	PL
T7	Number of veins	NV
T8	Single leaf weight (g)	WL
T9	Total number of leaves	TNL
T10	Content of gutta-percha (%)	GP
T11	Content of chlorogenic acid (%)	CA
T12	Osmotic potential at turgor loss	*ψ*_π_^0^
T13	Osmotic potential at full turgor	*ψ*_π_^100^
T14	Critical water saturation deficit	*RWD*_0_
T15	Relative bound water content at full turgor	*Ma*

**Table 2:** Variations and normal test for leaf-related traits in *Eucommia ulmoides* Oliver.

Traits	Planting Models ^a^	Tree Ages	Germplasm Number	Min.	Max.	Range	Mean ^b^	*SD* ^c^	CV ^d^ (%)	*p* Value ^e^
LL	L	1	48	192.4	272.7	80.3	232.8 A	17.0	7.3	0.7236
	A	8	56	92.7	221.3	128.6	147.1 B	27.3	18.5	0.2640
	A	9	56	107.0	160.3	53.3	134.5 C	11.4	8.5	0.9305
	A	10	56	120.7	209.3	88.6	147.6 B	14.7	10.0	0.1863
LW	L	1	48	93.0	138.2	45.2	115.2 A	11.3	9.8	0.6942
	A	8	56	39.0	94.9	55.9	63.8 B	12.5	19.6	0.1102
	A	9	56	46.2	80.1	33.9	60.8 B	8.3	13.7	0.0507
	A	10	56	51.2	93.3	42.1	66.9 B	8.0	12.0	0.1963
LA	L	1	48	12,314.8	22,737.1	10,422.3	17,877.1 A	2691.1	15.1	0.3470
	A	8	56	1971.4	12,558.9	10,587.5	5348.2 B	1664.0	31.1	0.1853
	A	9	56	3023.0	8191.3	5168.3	4893.3 B	1002.8	20.5	0.1302
	A	10	56	3383.1	11,749.5	8366.4	5905.9 B	1054.2	17.8	0.8026
LWR	L	1	48	1.62	2.46	0.84	2.07 B	0.19	9.1	0.9412
	A	8	56	1.61	3.21	1.60	2.35 A	0.29	12.3	0.2370
	A	9	56	1.80	2.84	1.03	2.24 A	0.24	10.7	0.3822
	A	10	56	1.60	2.81	1.21	2.25 A	0.26	11.4	0.3881
TL	L	1	48	0.43	0.59	0.16	0.51 A	0.04	7.7	0.6184
	A	8	56	0.14	0.40	0.26	0.25 C	0.05	18.9	0.0749
	A	9	56	0.18	0.36	0.18	0.26 BC	0.04	15.5	0.3698
	A	10	56	0.21	0.38	0.16	0.28 B	0.04	13.2	0.1244
PL	L	1	48	16.67	33.11	16.44	25.15 A	1.87	7.5	0.8569
	A	8	56	11.51	32.73	21.22	18.27 B	3.92	21.5	0.0681
	A	9	56	12.32	28.48	16.16	17.82 B	2.57	14.4	0.2036
	A	10	56	13.26	29.51	16.25	18.77 B	2.55	13.6	0.7481
NV	L	1	48	16.2	20.1	4.0	18.2 A	1.0	5.7	0.3619
	A	8	56	12.3	19.0	6.7	15.0 B	1.4	9.1	0.1447
	A	9	56	12.3	18.0	5.7	15.2 B	1.3	8.7	0.4919
	A	10	56	13.0	18.7	5.7	15.4 B	1.0	6.6	0.0721
WL	L	1	48	1.2328	2.6245	1.3918	1.8318 A	0.3144	17.2	0.3674
	A	9	56	0.1450	0.9157	0.7707	0.3766 B	0.1081	28.7	0.5583
	A	10	56	0.2027	0.9515	0.7488	0.4275 B	0.1110	26.0	0.5641
TNL	L	1	48	24	50	26	35 B	5	15.1	0.6885
	A	9	56	562	4060	3498	1656 A	550	33.2	0.1808
	A	10	56	347	3815	3468	1878 A	750	39.9	0.0843
GP	L	1	48	0.71	1.62	0.90	1.10 B	0.22	19.87	0.1229
	A	8	55	0.87	3.20	2.33	1.75 A	0.45	25.49	0.4512
	A	9	56	0.90	3.29	2.40	1.79 A	0.43	24.09	0.1148
	A	10	56	0.67	2.95	2.28	1.92 A	0.41	21.13	0.2740
CA	L	1	48	0.56	2.79	2.22	1.62 A	0.54	33.14	0.7486
	A	9	56	0.32	2.28	1.96	1.20 B	0.41	34.49	0.5308
	A	10	56	0.30	2.35	2.04	1.29 B	0.48	37.49	0.5547
*ψ*_π_^0^	L	1	48	13.06	16.81	3.75	15.09 A	0.92	6.07	0.1170
	A	10	50	12.08	16.91	4.83	14.55 B	0.79	5.46	0.0754
*ψ*_π_^100^	L	1	48	7.98	9.99	2.01	9.09 A	0.51	5.64	0.0647
	A	10	50	7.20	9.77	2.57	8.44 B	0.56	6.62	0.9538
*RWD*_0_	L	1	48	6.32	14.27	7.94	9.66 A	1.71	17.69	0.2198
	A	10	50	4.33	11.50	7.17	7.63 B	1.65	21.59	0.4522
*Ma*	L	1	48	0.61	0.85	0.24	0.75 B	0.05	6.25	0.3185
	A	10	50	0.71	0.89	0.18	0.82 A	0.04	5.52	0.2169

^a^ “L” indicates the Leaf-oriented cultivation model, “A” indicates the Arbor forest model. ^b^ The same letter for the same trait indicates a non-significant difference, while different letters indicate an extremely significant difference at “*p* < 0.01” level, according to Tukey HSD. ^c^ *SD* indicates standard deviation. ^d^ CV indicates coefficient of variation. ^e^ *p* value indicates the significance level of the normal distribution test. The same is below.

**Table 3:** Variance analyses of leaf-related traits in different planting models of *Eucommia ulmoides* Oliver.

Traits	Planting Model				Genotype			
	Df	MS	*F* Value	*p* Value	Df	MS	*F* Value	*p* Value
LL	1	828,917.83	1499.442	0.000	55	1855.53	3.356	0.000
LW	1	264,342.54	2093.847	0.000	55	588.84	4.664	0.000
LA	1	15,420,000,000	3019.958	0.000	55	16,918,968.05	3.314	0.000
LWR	1	3.030	35.737	0.000	55	0.359	4.229	0.000
TL	1	4.667	1089.642	0.000	55	0.004	0.899	0.678
PL	1	5023.425	495.826	0.000	55	91.702	9.051	0.000
NV	1	922.112	244.045	0.000	55	8.953	2.370	0.000
WL	1	117.398	1819.325	0.000	55	0.167	2.586	0.000
TNL	1	309,741,032.2	742.662	0.000	55	1,541,740.43	3.697	0.000
GP	1	43.648	415.807	0.000	55	0.857	8.162	0.000
CA	1	8.455	36.671	0.000	55	0.968	4.198	0.000
*ψ*_π_^0^	1	17.075	7.829	0.006	46	2.296	1.053	0.391
*ψ*_π_^100^	1	26.263	33.707	0.000	46	1.015	1.303	0.107
*RWD*_0_	1	815.416	44.351	0.000	46	16.858	0.917	0.627
*Ma*	1	0.635	50.352	0.000	46	0.012	0.913	0.634

Df: Degrees of freedom; MS: Mean square.

**Table 4:** Association analyses among leaf-related traits in different planting models of *Eucommia ulmoides* Oliver.

	LL	LW	LA	LWR	PL	NV	WL	TNL	GP	CA
LL	1	0.498 **	0.724 **	0.196	0.110	0.178	0.457 **	−0.296 *	0.277	−0.449 **
LW	0.701 **	1	0.933 **	−0.740 **	0.215	0.187	0.801 **	−0.211	0.006	−0.399 **
LA	0.873 **	0.940 **	1	−0.496 **	0.185	0.213	0.797 **	−0.279	0.125	−0.467 **
LWR	0.133	−0.604 **	−0.333 *	1	−0.151	−0.111	−0.545 **	−0.019	0.191	0.090
PL	0.387 **	0.127	0.234	0.289 *	1	0.218	0.229	−0.134	0.057	−0.168
NV	0.356 **	0.413 **	0.426 **	−0.155	0.236	1	0.055	0.026	0.108	−0.017
WL	0.447 **	0.462 **	0.524 **	−0.132	0.071	0.200	1	−0.080	0.153	−0.363 *
TNL	−0.023	0.118	0.062	−0.233	−0.201	0.078	−0.035	1	0.267	0.289 *
GP	−0.226	−0.223	−0.204	0.050	−0.152	0.139	−0.011	0.137	1	−0.315 *
CA	−0.174	−0.203	−0.181	0.068	−0.219	−0.015	−0.079	0.157	0.240	1

Association analyses among leaf-related traits in the Leaf-oriented cultivation model and Arbor forest model are, respectively, above and below diagonal. “*” and “**” indicate that the degree of correlation is significant (*p* < 0.05) or extremely significant (*p* < 0.01) among traits.

**Table 5:** The criterion of leaf-related traits in *Eucommia ulmoides* Oliver.

Traits	Planting Models	Grade		
		1 (Worse)	2 (Medium)	3 (Better)
LA (mm^2^)	L	≤16,465	16,465–19,230	≥19,230
	A	≤4730	4730–6035	≥6035
TNL	L	<32	32–37	≥37
	A	<1430	1430–2100	>2100
GP (%)	L	<1.00	1.00–1.20	>1.20
	A	<1.60	1.60–2.05	≥2.05
CA (%)	L	<1.35	1.35–1.90	≥1.90
	A	≤1.00	1.00–1.50	≥1.50

## Data Availability

The data presented in this study are available on request from the corresponding author.

## Supplementary Materials

The following are available online at https://www.mdpi.com/article/10.3390/plants10112280/s1, Table S1: The grading standard of leaf-related traits of *Eucommia ulmoides* Oliver in different tree ages.
