# Supplementary material for: Trait Variations and Probability Grading Index System on Leaf-Related Traits of Eucommia ulmoides Oliver Germplasm
Source: Plants (Basel). 2021 Oct 25;10(11):2280. doi: 10.3390/plants10112280 (PMC8620490; doi:10.3390/plants10112280)
Supplement: Supplementary file 1 [file plants-10-02280-s001.zip › Table S1.pdf]

Table S1. The grading standard of leaf-related traits of *Eucommia ulmoides* Oliver in different tree ages.

Traits	Tree ages	Planting models[a]	Grading points					
			1st	Merge[b]	Adjustment[c]	2nd	Merge[b]	Adjustment[c]
LA	1	L	16,465.4	16,465.4	16,465	19,288.8	19,288.8	19,230
	8	A	4,475.3			6,221.2		
	9	A	4,367.2	4,731.8	4,730	5,419.4	6,033.2	6,035
	10	A	5,352.9			6,459.0		
TNL	1	L	32	32	32	37	37	37
	9	A	1,367	1,426	1,430	1,944	2,108	2,100
	10	A	1,485			2,272		
GP (%)	1	L	0.99	0.99	1.00	1.21	1.21	1.20
	8	A	1.52			1.99		
	9	A	1.56	1.59	1.60	2.01	2.04	2.05
	10	A	1.70			2.13		
CA (%)	1	L	1.34	1.34	1.35	1.90	1.90	1.90
	9	A	0.98	1.005	1.00	1.42	1.48	1.50
	10	A	1.03			1.54		

[a] "L" indicates the Leaf-oriented cultivation model, "A" indicates the Arbor forest model.

[b] Average the phenotypic values in the same planting model for the same trait.

[c] Adjust the grading points.
